# An Emotional Bias Modification for Children With Attention-Deficit/Hyperactivity Disorder: Co-design Study

**DOI:** 10.2196/36390

**Published:** 2022-12-09

**Authors:** Melvyn Zhang, Vallabhajosyula Ranganath

**Affiliations:** 1 Family Medicine and Primary Care Lee Kong Chian School of Medicine Nanyang Technological University Singapore Singapore Singapore; 2 Anatomy, Office of Medical Education Lee Kong Chian School of Medicine Nanyang Technological University Singapore Singapore Singapore

**Keywords:** emotional bias, cognitive biases, attention-deficit/hyperactivity disorder, ADHD, child psychiatry

## Abstract

**Background:**

Attention-deficit/hyperactivity disorder (ADHD) is one of the common neurodevelopment disorders. Children with ADHD typically have difficulties with emotional regulation. Previous studies have investigated the assessment for underlying emotional biases using the visual probe task. However, one of the significant limitations of the visual probe task is that it is demanding and repetitive over time. Previous studies have examined the use of gamification methods in addressing the limitations of the emotional bias visual probe task. There has also been increased recognition of the potential of participatory action research methods and how it could help to make the conceptualized interventions more relevant.

**Objective:**

The primary aim of this study was to collate health care professionals’ perspectives on the limitations of the existing visual probe task and to determine if gamification elements were viable to be incorporated into an emotional bias modification task.

**Methods:**

A co-design workshop was conducted. Health care professionals from the Department of Development Psychiatry, Institute of Mental Health, Singapore, were invited to participate. Considering the COVID-19 pandemic and the restrictions, a web-based workshop was conducted. There were 3 main phases in the workshops. First, participants were asked to identify limitations and suggest potential methods to overcome some of the identified limitations. Second, participants were shown examples of existing gaming interventions in published literature and commercial stores. They were also asked to comment on the advantages and limitations of these interventions. Finally, participants were asked if gamification techniques would be appropriate.

**Results:**

Overall, 4 health care professionals consented and participated. Several limitations were identified regarding the conventional emotional bias intervention. These included the nature of the task parameters, included stimulus set, and factors that could have an impact on the accuracy of responding to the task. After examining the existing ADHD games, participants raised concerns about the evidence base of some of the apps. They articulated that any developed ADHD game ought to identify the specific skill set that was targeted clearly. Regarding gamification strategies, participants preferred economic and performance-based gamification approaches.

**Conclusions:**

This study has managed to elucidate health care professionals’ perspectives toward refining a conventional emotional bias intervention for children with ADHD. In view of the repetitiveness of the conventional task, the suggested gamification techniques might help in influencing task adherence and reduce the attrition rates.

## Introduction

### Background

Attention-deficit/hyperactivity disorder (ADHD) is one of the common neurodevelopment disorders. Children with ADHD typically display symptoms such as hyperactivity, impulsivity, and inattention [1]. These individuals have inherent underlying difficulties with emotional regulation [2]. The advances in experimental psychology have led to better understanding of emotional biases and methods by which these biases could be modified [3]. Previous studies have reported that emotional biases are most prevalent among those with the combined subtype of ADHD. Children with this subtype tend to have difficulties in comprehending the emotional states of others and recognizing facial emotions and emotional cues [2]. Previous studies have described how the visual probe or dot probe task is applied in the assessment and potentially in modifying these biases. In the visual probe task, individuals are presented with 2 stimuli on the screen simultaneously in the assessment phase. The presented stimuli are images showing different emotional cues, for example, an angry face or a neutral expression. The set of stimuli would disappear, and a probe would replace either stimulus. Individuals are required to indicate the position of the probe as rapidly as possible. For purposes of assessment, individuals may have positive emotional biases when they respond more readily to probes that replace the emotional stimulus instead of the neutral stimulus. For bias modification, the contingency of which stimulus is being replaced could be altered in a way that is similar to the application of the visual probe task for other psychiatric disorders [3]. Although there have been previous studies examining the effectiveness of cognitive bias modification for anxiety and depression among children and adolescents with other psychiatric disorders, for example, anxiety and depression [4,5], pioneering studies have been conducted in exploring emotional biases in the same population. In a recent study by Cremone et al [3], the authors reported the existence of emotional biases among children and adolescents with ADHD, and the amount of sleep affected the magnitude of the underlying emotional biases.

Although the visual probe task appears to be a relatively simple task to administer for the measurement of underlying emotional biases, and potentially for modifying biases, it is not without its inherent limitations. The numerous repeats that an individual needs to complete make such an intervention laborious. In recent years, serious games and gamification technologies have been considered for conventional psychological interventions [6,7]. It is believed that the use of these technologies would enhance engagement in the short and long term, promote self-empowerment, and improve existing skills [6]. As highlighted by Zhang et al [8], gamification, when applied to cognitive bias modification interventions, could help to reduce the repetitiveness of the game and increase motivation to train. As evident from previous study by Zhang et al [9], which involved conducting a series of participatory action research workshops involving health care professionals and patient service users, gamification could be used to address the limitations of the visual probe task and enhance the conventional task.

More recently, regarding ADHD, there have been further studies examining the evidence base of existing games for individuals with ADHD and the potential of gamification for serious games targeting individuals with ADHD. Jiang et al [10] conducted a scoping review of the currently available mobile games on several databases, and they reported that most of the existing games were focused on managing individuals with symptoms and less so on symptoms and diagnosis. Although the studies have shown an improvement in the performance of the children across the interventions, these studies were limited in several aspects, such as the inclusion of a small sample size and lack of a control group for comparison, and these factors influence the overall effectiveness of the study [10]. Nonetheless, the 19 studies highlighted the potential of considering a serious game approach when conceptualizing new interventions, and they also revealed that the most common techniques were that of having participants to respond to various cues, remembering details, or making association between different entities [10]. In another recent study by Sujar et al [11], the authors, having synthesized the evidence from literature, reported that future games that are designed for individuals with ADHD ought to consider the following aspects: the underlying mechanics of the game ought to be based on some form of cognitive exercise and therapeutic strategies that may be helpful include having levels of difficulties, a motivational element, time constraint for the task one has to undertake, and some form of reinforcement [11]. The findings reported by Sujar et al [11] are consistent with the aims and objectives of this study, which, as elaborated subsequently, aimed to seek the perspectives of health care professionals as an integral step in co-designing an intervention to modify emotional biases in children.

### Objectives

There has also been increased recognition of the potential of participatory action research methods and how it could help to make the conceptualized interventions more relevant [12]. Therefore, the aim of this study was to use such a method in refining the conventional emotional bias visual probe task for children and adolescents. The primary aim of this study was to collate health care professionals’ perspectives on the limitations of the existing visual probe task and to determine if gamification elements were viable to be incorporated into an emotional bias modification task. We sought to answer the following research questions: (1) What were health care professionals’ perspectives on the conventional emotional bias task? (2) What were health care professionals’ perspectives on existing gaming interventions for ADHD? (3) Would gamification be appropriate, and if appropriate, what strategies could be used?

## Methods

### Study Design

Principles of participatory action research were used for this study. A co-design workshop was conducted, and relevant key stakeholders (ie, health care professionals) were invited. Only health care professionals were invited, as they had knowledge of the psychiatric conditions and were best able to advise how the task could be modified, while adhering to the evidence base.

### Study Setting

Health care professionals from the Department of Development Psychiatry, Institute of Mental Health, Singapore, were invited to participate. A diverse group of health care professionals was invited, and it included both psychiatrists and psychologists. We had originally planned to recruit occupational therapists also, but none of them expressed an interest in participating in the study.

### Sample Size

On the basis of our previous protocol [13], we planned to recruit 8 participants. We managed to recruit 50% (4/8) of the projected participants for the focus group. However, there was a reduction in the number of participants recruited, mainly in part owing to the ongoing COVID-19 pandemic and the difficulties for individuals to commit to research studies as they have other clinical or administrative roles.

### Details of the Co-design Workshops

Owing to the current COVID-19 pandemic and local governmental restrictions, we applied for the conduct of the workshop via the web. Therefore, all participants (4/4, 100%) who have had expressed interest were contacted separately to sign the informed consent form. The participants also had to complete a baseline demographic questionnaire individually. This questionnaire collated information regarding age, sex, and years of experience in treating children and adolescents with psychiatric disorders. Upon completion of the questionnaire, the principal investigators liaised with each of the participants separately to identify a common time for conducting the web-based workshop.

**Figure 1:**
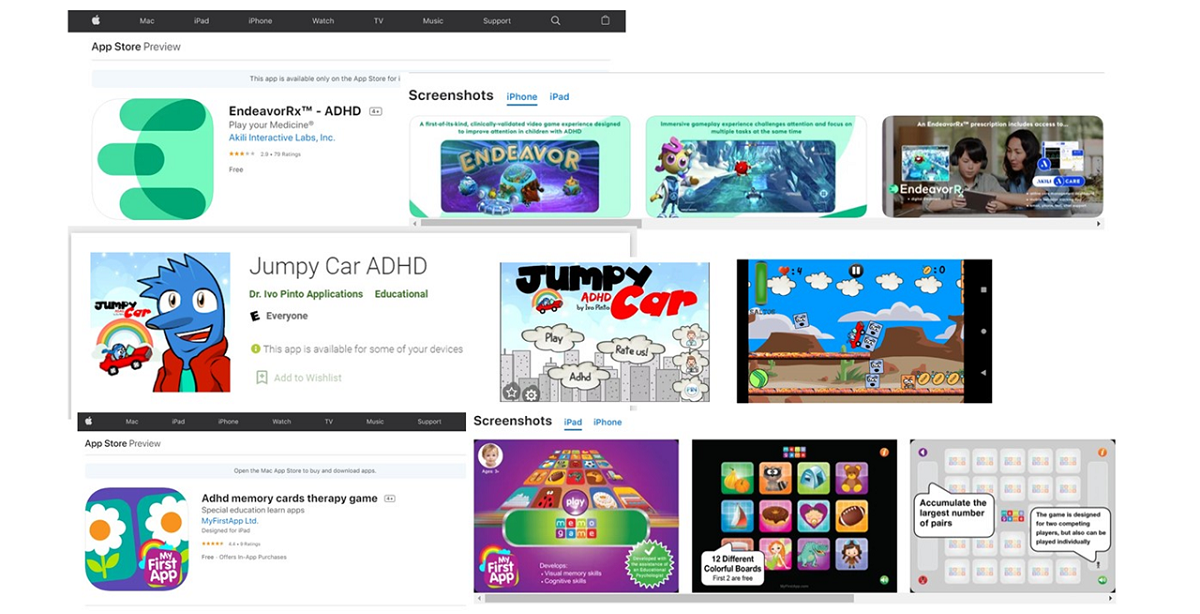
Examples of games shown to participants.

The workshop was subsequently conducted on May 19, 2021, via the web. Both principal investigators facilitated the workshop and recorded the field notes. All participants (4/4, 100%) were informed of the study’s rationale and the session’s specific objectives. Participants were also informed that their responses and comments were confidential and that the session would be audio-recorded. All participants (4/4, 100%) were given participant numbers to identify themselves, to provide further anonymity. First, participants were shown an example of the visual probe task paradigm that has been traditionally used for cognitive bias modification. The example that participants viewed was based on the previous study by Zhang et al [9]. In that study, the specific nature of cognitive bias modification was that of attention bias modification, and it was applied to individuals with addictive disorders. Then, participants were shown how the traditional visual probe task paradigm is modified to become an emotional bias task paradigm (by modifying the visual cues presented). Then, they were asked to identify limitations and suggest potential methods to overcome some of the identified limitations regarding the emotional bias modification intervention. Subsequently, participants were shown examples of existing gaming interventions in published literature and commercial stores. Figure 1 provides an overview of some of the games that were shown to the participants.

They were asked to comment on the advantages and limitations of these interventions. Finally, participants were shown a list of gamification techniques, and each of the techniques was explained to them. The list of gamification techniques was based on the previous study by Hoffman et al [14]. The list of gamification techniques is described in Table 1, and this has also been published in the previous study by Zhang et al [9]. They were asked if the inclusion of such techniques would be appropriate.

**Table 1:** Gamification techniques that were shown to participants.

Gaming approach			Description
**Economic gamification techniques**			
	Marketplace and economies	Providing gamers with a web-based currency that allows them to deal in the game	
	Digital rewards	These include badges, game currency, game points, web-based goods, and powers or abilities	
	Real-world prizes	Provides gamers with options to exchange in-game credits for real-world prizes such as vouchers or other forms of goods and services	
**Social gamification techniques**			
	Avatar	Allows individuals to choose a web-based character to represent oneself	
	Agent	A web-based character that guides or provides instructions to the user	
	Competition	Allows individuals to compete with other players or with each other	
	Teams	Game that involves several individual players, allowing them to interact and form relationships	
	Parallel communication systems	Allows individuals to communicate with one another	
	Social pressure	Ability of game to pressurize individuals to perform in certain task, so that they will be invited to subsequent events	
**Performance-orientated techniques**			
	Feedback	Spoken, visual, or auditory feedback about user’s performance	
	Levels	Information on the stage of a game one has attained	
	Secondary game objectives	Secondary goals that reward the player upon completion	
	Ranks of achievement	Measurement of character development	
	Leaderboards	Allows for comparisons with other players	
	Time pressure	Predetermined time limits for task completion	
**Embedding-focused techniques**			
	Narrative context	A storyboard or stories that guide the development of the character	
	3D environment	3D models of objects that parallel the real world	

### Data Analyses

The descriptive data (demographics) were summarized as means and SDs. The audio recording obtained from the workshop was transcribed verbatim. One of the principal investigators, MZ, performed the first transcription and developed the coding frame. To ensure the reliability of the coding frame that was adopted, two authors (MZ and RV) reviewed the transcripts and discussed the coding frame. This ensured that the process of intercoder consensus was adhered to. Codes that were identified were classified into categories and then reorganized into themes. The themes that were generated were subsequently reviewed and further refined. The underlying methodology used was in accordance with the previous recommendations by Braun and Clarke [15]. NVivo (version 12.0; QSR International) [16] was used for thematic analysis.

### Ethics Approval

This study was approved by the ethical review board of the Nanyang Technological University Singapore (approval number IRB-2020-03-058). Informed consent was obtained from all the participants, and they were also informed that they could withdraw from the study at any time.

### Data Management

No participant-related identifiers were captured on the hard-copy questionnaires. All the completed hard-copy forms and informed consent forms were stored in a secured facility. The audio recordings of the workshop were transferred to a local secured computer for storage, and the original recording was deleted from the recording device. The password of the local computer was changed frequently, and only the principal investigators were able to access the files. All the records and audio recordings will be maintained for a period of 6 years following the completion of the study. All participants were provided an inconvenience fee for their time and effort in participating in the study.

## Results

### Demographics

A total of 4 health care professionals consented and participated in the workshop, which was conducted on May 19, 2021. Of the 4 health care professionals, 1 (25%) was a child and adolescent psychiatrist and the remaining 3 (75%) were psychologists. The mean age was 43.5 (SD 5.74) years. Of the 4 participants, 1 (25%) was a man and the remaining 3 (75%) were women. The mean years of experience was 14.5 (SD 4.43) years, ranging from 10 to 20 years. Table 2 provides an overview of the baseline demographics of the participants.

**Table 2:** Overview of the baseline demographics of the participants (N=4).

Participant number	Age (years)	Nationality	Sex	Experience (years)	Race
1	52	Singaporean	Male	20	Chinese
2	42	Singaporean	Female	16	Chinese
3	40	Singaporean	Female	10	Chinese
4	40	Singaporean	Female	12	Indian

### Phase 1

In phase 1, the participants identified several limitations regarding the nature of the conventional visual probe task used to assess emotional biases. These limitations were related to the nature of the task parameters, included stimulus set, and other factors that could affect the accuracy of responding to the task. Participants also made recommendations regarding how the task could be improved. Textbox 1 provides a summary of the verbatim comments of the participants for each of the identified themes.

**Themes related to the nature of the conventional visual probe task.**

**Theme 1**

- Limitations—Issues related to task parameters
- Verbatim comments
  “I suppose, theoretically, it sounds alright. I am a bit concerned about the speed at which the task goes.” [Participant 2]
  “The design of the task, flashing multiple images in quick succession, can be distressing and could cause fatigue.” [Participant 1]
  “I think the number of trials is ok. I think the speed I am not comfortable with.” [Participant 3]
  “I am wondering about the practice trial. There are 8 trials. Do they go at the same speed as the actual 32? I don’t know whether existing emotional bias trials. When it is applied to children and adolescent. Could the speed be moderated? Or you could do half half. 8 which is lower speed to aid understanding the next 8 is at actual speed.” [Participant 2]
  “I think just now my mind was a blank at the speed. Wow. What was there. Even I know what the instructions were and what I am supposed to do, I was not able to process when I saw the program.” [Participant 4]

**Theme 2**

- Limitations—Stimulus set
- Verbatim comments
  “The other thing I wonder whether erm. In terms of the pictures, would it make a difference if you are used Asian versus Caucasian pictures. Would it make a difference?” [Participant 4]
  “I am just wondering about the size of the photos. Some of the photos were quite big. Because the speed was quite fast, all I saw was a blur of colours. For this current picture, there was white space. For the previous example, all I saw was a blur of colours.” [Participant 4]
  “I just want to echo the other respondent. If you could frame the picture like in the slide now, it homes the picture better. Rather than big white background. Black border, framing into a small visual field, might be more user-friendly.” [Participant 1]

**Theme 3**

- Limitation—Other confounders that may affect the accuracy of the task
- Verbatim comments
  “Whether the speed would trigger impulsivity and so. Perhaps there is a confounder. Impulsivity or desire to put in an answer. Might confound with reaction time in measuring attentional biases. This is my gut feel. I do not know the research behind it.” [Participant 2]
  “Children with ADHD do have comorbidities. Some of them have processing speed abnormalities. Could confound. Going back to your inclusion criteria you might want to think about.” [Participant 3]
  “I am concerned about impulsivity. Because it is going so far, I suspect that it is still going to be quite novel for kids with ADHD. But novelty aside, I think if they don’t know what they are doing, they just guess. You probably get a big bias which is not what you are looking for.” [Participant 2]

**Theme 4**

- Recommendations pertaining to presentation of the stimulus set
- Verbatim comments
  “For this current picture, there was white space. For the previous example, all I saw was a blur of colours.” [Participant 4]
  “I just want to echo the other respondent. If you could frame the picture like in the slide now, it homes the picture better. Rather than big white background. Black border, framing into a small visual field, might be more user-friendly.” [Participant 1]

### Phase 2

In this phase, participants were asked to provide their perspectives about the existing ADHD games. Several themes arose from the discussion. Participants highlighted that the games needed to be age-appropriate and clearly explain the specific skill sets that were targeted. Participants also raised concerns regarding the scientific evidence base for some of the presented games. Participants also highlighted issues with existing games regarding their novelty and stated that the consideration of novelty was important when implementing games for individuals with ADHD.

Textbox 2 provides a summary of the verbatim comments of the participants for each of the identified themes.

**Verbatim comments of the participants for each of the identified themes.**

**Theme 1**

- Being age-appropriate
- Verbatim comment
  “Erm. I think these games appear to cater to different age groups. The animations. Some of them appear more sophisticated than other. For example, the first game appeared more sophisticated and complex. The third one seems to cater to younger population. So I think we need to be aware of how appealing it is to each age group of the child is in.” [Participant 1]

**Theme 2**

- Skill sets targeted
- Verbatim comments
  “The second one, Jumpy car seems to be psychoeducational. Comprehensive package of game and psychoeducation. For psychoeducation, the second games seemed to be having more of that. For the other two, it seems to focus on skills and skills building. That is my first and most immediate observations of these games.” [Participant 1]
  “The other thing I noted for the second one. Between the games they give you psycho education.” [Participant 4]
  “I do quite like Jumpy car ADHD. I don’t have ADHD but I thought it is a good tool to engage them and helping them to understand what ADHD is. Of course, I have checked out the language and whether it is child friendly. Not sure about this memory card game. not sure what it was targeting about.” [Participant 2]
  “This is number 3. I also like the jumpy car and I do have ADHD. I can’t download it. Just had a thought. Just now you mentioned about combining. Jumpy car just seemed to get through all the distractors and getting to the point destination. Wondering if you could combine that frustration with not being able to cross the hurdles. So, the distractions. When they cannot cross the distractions, they are somehow emotional. So, one way may be is to add on to that. Does that make sense? Instead of getting from A to B, that it. Just to get rid of the distractor or to recognise the distractions. Teach them to recognise their emotions. A cognitive component to it.” [Participant 3]
  “Just wondering about the objective of doing emotional bias modification game for children with ADHD. So, what are we hoping the kids with kids with ADHD would become after the end of the game?” [Participant 2]
  “Mine is along the same line. What is the game setup to do. Is it going to be specific for emotional biases, capturing emotional biases. So the game, how it is designed, needs to tackle that. It needs to be valid and also be specific. Intending to assess and moderate. Teach skills on how to minimise the emotional biases. Some of the games shared seemed to focus on different skill sets. Jumpy car was to ignore distractions. So there was actually no emotion. Not much emotional component to that. And then the third one. About ADHD memory game. More like on working memory. They may be. My sense is that they are targeting different deficiencies seen in ADHD. I think the game design would have to include situations in which they could trigger emotional reactions. Frustrations or disappointments. We do understand from past research. The brains of the child is different from adult. Different parts of the child brain is being activated when given a similar task, compared to adult brain. And there is also less. They also have more problem, for teenagers especially, in recognising neutral emotions, they tend to over interpret neutral faces as anger. If you want to have a game that addresses emotional biases, then we have to also take on this consideration as well. The game must be specific to you know. To target emotional situations for example.” [Participant 1]
  “I think I may have misunderstood what you have asking. I think it is viable to convert to game. But like what participant 1 said, it has to be a situation that evokes the emotions. It is rather challenging now to kind of. Scope it in games, especially for our gen Z population right now. Just having situations taking example, game I, this is not going to really elicit that. Back to the previous point where you presented with ADHD games. Different ADHD games train different functions. Memory game would be working memory. Jumpy car would be impulsive control, or distraction. If you could add that emotional component to existing game. Would it be adding another layer? That is what I am trying to say? They run into situations and they do react. If there is a psycho edu component, what you can do about it and what you could do about it. Do I make sense now?” [Participant 3]
  “I am also wondering how the kids can play this and how this can be translated to real life. Because a lot of times they play the game, the skills stay in the game. When I am out in the real world, everything in the game, the skills I learnt does not apply in the real world. Some way for the kids to know that the skills they applied can be applied to the real world. Maybe like a short film clip or someone demonstrating that skills can be used. Kids should know that this is something that can be used in the real world.” [Participant 4]

**Theme 3**

- Scientific evaluations
- Verbatim comment
  “I suppose I am wondering that in terms of games that have been subjected to research evaluations, in terms of the follow-up period, whether the improvement have been sustained. I know that endevour RX has some trials back. Data sustained attention. What sort of ratings are being used during the trial. We have a few of attention improvement games. We also try to include blinded and objective rating for example measuring brain waves to measure whether there are actual changes. I wonder about the follow-up period, sustainability in terms of improvement and whether there are any objective measures for these kinds of game. Ditto for those who does not have any research behind.” [Participant 2]

**Theme 4**

- Novelty and motivation to use
- Verbatim comments
  “The other thing I noted for the second one. Between the games they give you psycho edu. If there is a skip function, I suspect that a lot of kids would skip and go to the game. They might not listen to it at all. If it is too repetitive and increasing in difficulty, kids would just give up especially when the novelty wears out. They try out the first few stages, but it is getting tougher, I just skip to something else.” [Participant 4]
  “I am going to start that I don’t know enough about EB games. I wonder about. If we have a monkey going down the supermarket, ignoring negative emotional faces. number 1. I am thinking about novelty. After a while ADHD kids would lose interest. Number 2 is it going to have an impact on emotional dysregulation. Games are seemingly more friendly platform to engage kids with ADHD versus paper and pencil. There is definitive value in gamifying it. Effectiveness whether it does modify EB. How do we make it more interesting? Stage base. Can’t be monkey going down the aisle. They are going to get bored soon.” [Participant 3]

### Phase 3

In this phase, the facilitator explained to the participants more about the common gamification strategies that have been used. Then, participants were asked to select the most appropriate gamification strategies that could be applied to the conventional emotional bias modification task. Strategies such as economic and performance-based gamification were deemed to be more appropriate.

Textbox 3 provides a summary of the gamification techniques that the participants have selected and their justifications for their selection.

**Gamification techniques selected by the participants and their justifications for their selection.**

**Theme 1**

- Economic approaches
- Verbatim comments
  “Like in my work with children and parents with ADHD. Often talk about how children with ADHD are motivated by 3 different factors. First novelty, second interest and third competition. I am looking at the gaming approaches. Things like having economic gamification approach, having digital rewards or real-world rewards might be one.” [Participant 2]
  “I think digital rewards will be helpful. A lot of kids have problems with delayed gratification. Some form of immediate reward would be helpful.” [Participant 1]
  “I agree with what has been said as well. And also, I think the kids are motivated when they can buy some. They like to browse the store and see what they can buy.” [Participant 4]
- “I have been taking about novelty.
  Real world prizes
  might be more relevant. After a while, digital rewards might not be too attractive for them.” [Participant 2]

**Theme 2**

- Performance-based approaches
- Verbatim comments
  “The other thing that I am looking at is performance orientated gaming approach. Where there is some sort of competition. Mindful that it is not too hard for the child. That the child is being ranked very far below. Competition, having that interest, economic gamification. Those two are ones to go with.” [Participant 2]
  “A lot of local kids have a competitive edge as well. Having a game, allowing them to level up. A lot of reinforcement, feedback that reinforces the child behaviour would fare better.” [Participant 1]
  “I agree with 1 and 2. The performance one, like what 2 said. Ranking low might result in them losing motivation.” [Participant 3]

**Theme 3**

- Inappropriate strategies
- Verbatim comment
  “I have been taking about novelty. Real world prizes might be more relevant. After a while, digital rewards might not be too attractive for them. You asked about which techniques are not suitable. I do wonder about the team part. Where they play with other people or interact with other community. Some of the children I work with have bad experiences gaming in teams, where they have social conflicts. We do see some form of social conflicts in children with ADHD. That a question mark for me. There are ones who go onto form close knitted communities?” [Participant 2]

**Theme 4**

- Risk of gamification
- Verbatim comments
  “I generally do not have any concerns about gamification as treatment modality. My concern is how accessible it is and its affordability as well. I think gamification has the benefit of allowing participant to play the game at his own time, under the supervision of caregiver or parent. Therapy could take place outside of the clinics. Probably more about accessibility and affordability. Probably more research studies to show that It works over the long term.” [Participant 1]
  “I would always caveat it. It must be supervised and moderated by adults. ADHD population has problem moderating screen time.” [Participant 2]

## Discussion

### Principal Findings

This is the first study that has explored the use of participatory action research as a research design to refine the conventional emotional bias modification task. This study complements the ongoing research that has been highlighted in the *Introduction* section, which demonstrated the presence of emotional biases among children and adolescents with ADHD. In our study involving health care professionals, several limitations were identified regarding the conventional emotional bias intervention. These included the nature of the task parameters, included stimulus set, and factors that could have an impact on the accuracy of responding to the task. After examining the existing ADHD games, participants raised concerns about the evidence base of some of the apps. They articulated that any developed ADHD game ought to clearly identify the specific skill set that was targeted. When health care professionals were eventually asked to select gamification strategies that could improve the conventional intervention, they preferred economic and performance-based gamification approaches.

Some of the identified limitations of the existing conventional visual probe paradigm used for the assessment of emotional biases are congruent with the findings of previous studies. In our study, health care professionals identified limitations pertaining to the task parameters, particularly that which pertains to the speed of presentation of the stimulus set. This is congruent with the previous co-design workshop, by Zhang et al [9], involving health care professionals and patient participants, who similarly reported that the presentation of the stimulus was very rapid. In that study, the participants recommended a lengthy stimulus presentation time to allow them to process the set of stimulus images [9]. On the basis of the review by Zhang et al [9] about the paradigms for the visual probe task as applied for addictive disorders, it would be of important to have stimulus presented for both short and long stimulus intervals. This allows for assessing both attentional processes, namely, initial orientation and delayed disengagement. This should be considered when developing the task paradigm for assessing emotional biases among adolescents. Another limitation that was identified pertains to the nature of the images presented. Health care professionals recommended for standardization in the size of the images, whether images are presented with or without borders, and how much these presented images contrasted against the background. These identified limitations are crucial and need to be considered when designing the next iteration of apps that assess emotional biases.

One of the concerns raised by health care professionals about existing ADHD games was whether these games were based on validated frameworks and adhered to the evidence base. In our workshop, we presented participants with examples of ADHD games that are commercially available, some of which have been previously evaluated. In a recent review by Penuelas-Calvo et al [17], they examined video games for the assessment and intervention of individuals with ADHD. A total of 22 papers were identified, and they reported that the existing tools were influential in establishing whether individuals do have attentional issues as compared with the control. Unfortunately, the review was published after we conducted our focus group. Otherwise, the data obtained from the review would have helped to advance the discussion. Another concern highlighted during our focus group was the need for games to be specific in terms of identifying the skill sets they sought to target. In the examples we shared, it appeared that some of the commercially available apps were specific in terms of what they wanted to develop. Penuelas-Calvo et al [17], in their examination of the literature, reported that most of their identified video games have been developed based on previously validated tasks, for example, the performance task by Corner and the go–no-go task. Regarding video games that were interventional in nature, most of them focused on cognitive training, such as improving executive functioning, attention or working memory, reaction time, cognitive flexibility, or motor ability. The responses obtained from our focus group and the findings by Penuelas-Calvo et al [17] further highlight the importance of computerized interventions on previously validated evidence-based tasks. It is also important to be mindful that when adapting the task to a computerized intervention or even a video game, the mechanism of the conventional task should not be altered.

Our participants also reported various gamification strategies that may benefit our emotional bias assessment task. Previous study has justified the importance of considering gamification strategies, given that they help with the improvement of engagement rates and reduction of dropouts [6]. The perceptions of our team of participants in the co-design workshop indicated that repetitive activities in the game with increased difficulty levels would potentially affect the performance, continuity, interest, and novelty of the game. Novelty, user-friendliness, and interactive game design concepts should be considered, so that the ADHD games can be made engaging effectively. One of the critical perspectives shared during the workshop was the need for clear instructions and directions in the gameplay. Otherwise, it is likely that children with ADHD may be frustrated with the game. Regarding the gamification strategies suggested, leader boards, digital rewards, and real-world prizes could engage the users and allow them to play the intervention multiple times. Our participants also advocated the consideration of economic approaches, given that they offer individuals a tangible, immediate reward. Despite these suggestions, we need to acknowledge that one of the major limitations of our study was that we have not considered the perspectives of children themselves and those of their caregivers. It is important to understand the perspectives of children, so that the intervention could be personalized to their needs. Although we agree with the previous recommendations by Penuelas-Calvo et al [17] that health care professionals should collaborate with computer engineers, we feel that apart from collaborating with an engineer to ensure a high-quality app that resembles the quality of commercial apps, it is far more critical to ensure that the app is personalized to the needs of the patients.

The main strength of this study was the use of co-design methods in the refinement of an existing evidence-based paradigm for the assessment of emotional biases. This helped to ensure that the eventual design is based on evidence but meets the potential needs of end users. This study also presented examples of existing ADHD apps to participants. In doing so, we were able to allow the participants to have a better in-depth understanding of existing apps and identify issues and limitations with existing apps. Despite these strengths, our study had several limitations. Although we initially planned to recruit a total of 8 participants, we managed to eventually recruit only 4 (50%) participants. The COVID-19 pandemic affected our ability to recruit participants, as they may have to attend to other clinical needs. In addition, although we had previously planned for a physical workshop, the COVID-19 pandemic prevented the execution of such a workshop owing to infection risks. Thus, we were limited to conducting a web-based workshop, which may have resulted in challenges among participants in responding. It would be ideal to also obtain insights from patient participants or their caregivers.

### Conclusions

This study has managed to elucidate health care professionals’ perspectives on the refinement of a conventional emotional bias intervention for individuals with ADHD. Taken together, gamification strategies could be applied to conventional emotional bias interventions. The findings from this study have implications on the subsequent studies seeking the personalization and gamification of such apps. Although we have sampled the perspectives of health care providers, it remains necessary to discuss these perspectives with the intended sample group as the next step, to ensure that the future conceptualized app will be consistent with their needs.

## Abbreviations

ADHD: attention-deficit/hyperactivity disorder

## Data Availability

All available data have been included in the manuscript.
